# Effects of a 12-Month Multicomponent Exercise Program on Physical Performance, Daily Physical Activity, and Quality of Life in Very Elderly People With Minor Disabilities: An Intervention Study

**DOI:** 10.2188/jea.JE20081033

**Published:** 2010-01-05

**Authors:** Naoto Taguchi, Yasuki Higaki, Shinichi Inoue, Hiromi Kimura, Keitaro Tanaka

**Affiliations:** 1 Department of Preventive Medicine, Faculty of Medicine, Saga University, Saga, Japan; 2 Department of Sports Science, Faculty of Culture and Education, Saga University, Saga, Japan; 3 Department of Community Health and International Nursing, Faculty of Medicine, Saga University, Saga, Japan

**Keywords:** exercise intervention, elderly, physical performance, quality of life, long-term care insurance system

## Abstract

**Background:**

Although studies suggest that exercise training improves physical performance and health-related quality of life (HRQOL) among elderly people, most of these studies have investigated relatively healthy persons. The objective of the present study was to determine the effects of a 12-month multicomponent exercise program on physical performance, daily physical activity, and HRQOL among very elderly people with minor disabilities.

**Methods:**

The subjects consisted of 65 elders (median age: 84 years) who were certified to receive long-term care in the form of support only or Level 1 care (the lowest level of care required); 31 were allocated to the intervention group and 34 to the control group. The intervention group participated in supervised exercises once a week for 12 months and in home-based exercises. The exercise program consisted of various exercises related to flexibility, muscle strength, balance, and aerobic performance.

**Results:**

After 12 months of exercise training, the intervention group had significant improvements in lower-limb strength and on the sit-and-reach test; these effects were not observed in the control group. The control group had significant decreases in grip strength, 6-minute walking distance, walking speed, and stride length; these decreases were not observed in the intervention group. No clear differences in HRQOL measurements or changes in physical activity were detected between groups.

**Conclusions:**

The 12-month multicomponent exercise program may effectively improve and maintain the physical performance of very elderly individuals with minor disabilities.

## INTRODUCTION

Japan has the longest life expectancy in the world, and the number of adults aged 65 or older is estimated to increase to around 38.5 million (36.5% of the total population) by 2040. Moreover, the number of adults aged 75 and over (the old-old) will exceed that of adults aged 65–74 years by 2020.^1^ Due to this situation, a public long-term care insurance system was started in April 2000 in Japan, which is the first and only insurance system in the world that provides help in the daily lives of elderly people with disabilities. After the implementation of this system, however, the number of elderly people who were certified for long-term care grew from 2.2 to 4.1 million (1.9 fold) in only 5 years. In particular, people requiring limited care (support only or Level 1 care) increased steeply (2.4 fold), and accounted for half the total certified population in April 2005. Most people requiring this limited support or care have been classified with disuse syndrome (inactivity syndrome).^2^

To slow the progression from minor disability to moderate or severe disability, the public long-term care insurance system was transformed to a more prevention-oriented program in April 2006, because a demographic shift in the severity of disabilities in elderly persons would financially overburden the system. The new system emphasizes improvement of physical fitness, nutrition, and oral function, as well as the prevention of withdrawal, dementia, and depression. Under this revised program, a physical exercise program has been implemented as an optional service that targets those who require only limited support or care.^2^ The actual content of the exercise program is determined by local health care providers, who are tasked with developing an effective and safe training program that is appropriate for their particular setting.

There is limited evidence on the effects of exercise training for very elderly people with disabilities. Two research groups have examined the effects of an exercise intervention, lasting approximately 12 months, among frail elders aged 70 years or older at facilities such as nursing homes^3^^–^^5^; one group reported that tai chi training, as compared to a wellness education program given to a control group, significantly improved the ability to stand up when sitting in a chair; it also improved body mass index, systolic blood pressure, resting heart rate,^3^ and the fear of falling.^4^ The second study demonstrated that a strength and flexibility program using simple, portable, and inexpensive equipment had a significantly more beneficial impact on the timed up-and-go test, physical performance test, mini-mental status examination, and the Berg balance scale than did recreational therapy for a control group.^5^ In contrast, a 4-month randomized trial of 1-on-1 physical therapy (ie, exercises emphasizing range-of-motion, strength, balance, transfer, and mobility) for very frail nursing home residents showed no significant improvements for any of the outcomes, except mobility, when compared to a control group with no exercise intervention.^6^ This might have been due to the short intervention period. Thus, it is necessary to conduct longer intervention studies of very frail elderly people, and to include a control group who maintain their usual daily activities.

The purpose of this study was to determine whether a 12-month multicomponent exercise program for very elderly people with minor disabilities, including those who use day services (eg, receiving assistance in taking a bath, eating meals, and rehabilitation) offered by the public long-term care insurance system, can improve physical performance, daily physical activity level, and health-related quality of life (HRQOL).

## METHODS

### Subjects

**Figure 1.**
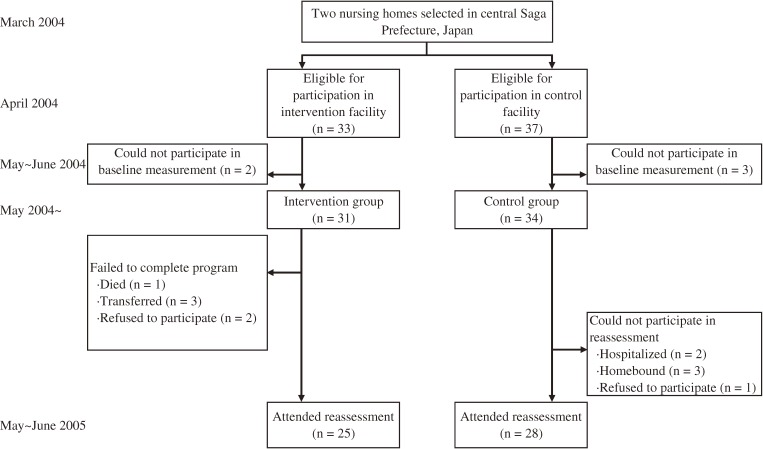
The trial profile

This prospective, nonrandomized, intervention study comprised an intervention group from one nursing home and a control group from another nursing home. The 2 nursing homes (the intervention facility in Taku City and the control facility in Ogi City) were located in central Saga Prefecture, in Japan. The facilities were selected because of their understanding of the study purpose. In April 2004, the subjects were selected from the users of day services in both nursing homes. The eligible subjects comprised elders certified to receive long-term care in the form of support only or Level 1 care. Level 1 care certification is granted when an elderly person experiences instability when standing up or walking and requires partial care in bathing and managing the household budget. In addition, the intervention group had to be able to visit the day service facility on Thursdays, when the exercise classes took place. The maximum number of subjects who could be accepted in the exercise class was approximately 30. A total of 33 elderly subjects (6 men and 27 women) aged 74 to 96 years met these criteria in the intervention facility. Next, we recruited an approximately equal number of control subjects, and attempted to ensure that their distribution by sex and the level of required care would be as close as possible to that of the 33 candidates for intervention. A total of 37 subjects (6 men and 31 women) aged 77 to 96 years were chosen from the control facility. Among the total of 70 candidates, 2 in the intervention group and 3 control subjects could not participate in the baseline measurements and thus were excluded. Ultimately, 31 subjects (6 men and 25 women) in the intervention group and 34 control subjects (6 men and 28 women) participated in the present study (Figure 1). All participants gave their written informed consent to participate in the study after being informed of all risks, discomforts, and benefits associated with the procedures to be followed in the study. The study protocol was approved by the ethics committee at the Faculty of Medicine, Saga University. This study was conducted under the aegis of the long-term care insurance system and was supported by a local organization that assisted in administering the system (the area-wide association of central Saga Prefecture).

### Intervention

From May 2004 through June 2005, the intervention group participated in a 12-month multicomponent exercise program, which comprised both supervised and home modules. The class-based module consisted of a weekly 90-minute supervised exercise session; there were 55 sessions in total. These sessions substituted for the functional training usually provided in day services. The exercise program consisted of various activities related to flexibility, strength, endurance, balance, aerobic performance, body awareness, and rhythm. Each session was led by a trained fitness instructor who was certified by the Aerobics and Fitness Association of America and had 13 years of experience in guiding exercise training. One of the authors was also available to support the instructor. The group was divided into several subgroups each comprising 5 to 6 participants, and 1 staff member from the facility supervised each subgroup. All exercises in the program were performed slowly to ensure the safety of the subjects, and emphasis was placed on good posture, as well as on social interaction and enjoyment. The supervisors did not force any participant to perform an exercise if he/she felt anxious about doing so. Each exercise session began with gentle movements while seated on a chair (approximately 10–30 minutes) to improve mobility of the upper and lower body, followed by the specific exercises described below and in Table 1. The session ended with a cool-down period on a chair (approximately 10–20 minutes), which allowed for relaxation and stretching. The intensity of each component exercise was estimated by using the guidelines of the American College of Sports Medicine^7^ and the compendium of physical activities.^8^

**Table 1.** Exercises included in the class-based exercise program

Period	Category	Intensity^a^	Type	Equipment	Content
Months 1–3	Chair-sitting	Light	Stretching and aerobic activity	Towel, ball	Movement of leg, trunk, and arm with music

			Strength activity	Rubber band, ball	Movement of shoulder, elbow, trunk, hip, knee, and foot

Months 4–6	Chair-sitting	Light	Same as above	Same as above	Same as above, except that the duration and intensity of aerobic and strength exercises were gradually increased on an individual basis

	Standing	Light-to-moderate	Strength activity	Chair, rubber band	Rise from chair, half-squats, rise to tiptoe position, knee raise, and hip extension

	Walking	Moderate	Aerobic activity	None	3–6 minutes in duration

Months 7–	Chair-sitting	Light-to-moderate	Same as above	Same as above	Same as above, except that the duration and intensity of aerobic and strength exercises were further increased

	Standing	Light-to-moderate	Same as above	Same as above	Same as above

	Balancing and combined exercises	Light-to-moderate		Ball, rubber band	Eg, (for balance) one-legged standing with chair as support, sitting on soft ball with stable posture

	Walking	Moderate	Aerobic activity	None	Prolonged duration (maximum 6 minutes)

^a^Light, around 2.0 metabolic equivalents [METs]; Moderate, around 3.0 METs (estimated from References 7 and 8).

During the first 3 months, all specific exercises were performed on a chair (1.5–2.5 metabolic equivalents [METs]). The exercises, which attempted to increase flexibility and aerobic capacity, were accompanied by music and began with continuous movement of the legs and trunk and intermittent movement of the arms. Then, strength exercises were performed with a rubber band, a ball, or the participant’s body weight. Specifically, these exercises involved movements of the shoulder (abductors, adductors, and rotators), elbow (flexors and extensors), trunk (flexors and extensors), hip (flexors, extensors, abductors, and adductors), knee (flexors and extensors), and foot (ankle dorsal and plantar flexors).

During the next 3 months, standing exercises using a chair as support (ie, rise from a chair, half-squats, rise to a tiptoe position, knee raise, and hip extension) were begun as strength exercises. In addition, the duration and intensity of aerobic and strength exercises in the above chair-sitting exercises were gradually increased on an individual basis. Walking for approximately 3 to 6 minutes was also added as an aerobic exercise. The intensity of each component exercise ranged from approximately 1.8 to 3.0 METs. After 6 months, the duration and intensity of the aerobic and strength exercises were further increased, and balance exercises (eg, 1-legged standing with a chair as support and sitting on a soft ball with a stable posture) and combined exercises were introduced.

The exercises in the home-based module were very similar to those of the in-class regimen. Participants in the intervention group received a weekly 1-page handout that illustrated and explained the physical exercises, which consisted of stretching and strength exercises using body weight and a towel. We recommended that the participants do the home exercises for 10 minutes every day except Thursdays, when the supervised exercise sessions were held. The intensity of the exercises was gradually increased, and dynamic and combined exercises were gradually introduced. Compliance with the home regimen was checked by examining the training diary that was completed by each subject.

No specific intervention was conducted for the control group.

### Measurement of physical performance, walking ability, and HRQOL

At baseline (May-June 2004) and 12 months later (May-June 2005), the subjects who had not dropped out were examined for body mass index, physical performance, walking ability, and HRQOL. Physical performance measurements included muscle strength (lower-limb and grip strength), balance (timed 1-legged standing with open eyes), flexibility (sit-and-reach test), and aerobic endurance capacity (6-minute walking distance). Walking ability measurements were estimated by analyzing movement, and comprised walking speed and stride length, as well as knee joint angle, trunk angle, and thigh angle upon heel contact. HRQOL measurements comprised the Instrumental Activities of Daily Living (IADL, 8 items, 0–8 points),^9^^,^^10^ Mini-Mental State Examination (MMSE, 11 items, 0–30 points),^11^^,^^12^ Philadelphia Geriatric Center Morale Scale (PGC-morale scale, 11 items, 0–11 points),^13^^–^^15^ Geriatric Depression Scale (GDS, 5 items, 0–5 points),^16^^–^^18^ Tokyo Metropolitan Institute of Gerontology Index of Competence (TMIG-IC, 13 items, 0–13 points),^19^ and the Falls Efficacy Scale (10 items, 10–40 points).^20^^,^^21^ For all HRQOL measurements except GDS, a higher total score indicated a higher capacity. For GDS, a higher total score denoted more severe depression.

Lower-limb strength (isometric knee extensor strength) was measured with the knee flexed to 90° (GF-300, YAGAMI Co., Ltd., Nagoya, Japan). Each subject sat on a pedestal with a strap placed around the leg just proximal to the ankle joint, and was asked to kick forward with maximal force. Grip strength was assessed with a digital squeeze dynamometer (YDM-110D, YAGAMI. Co., Ltd., Nagoya, Japan). For these 2 measurements, 2 test trials were completed on each side of the body. The final score was the average of the best right- and left-side scores. In timed 1-legged standing, each subject conducted 1 practice trial and 1 test trial on each side, and the average of the values from the right and left sides was used in the analysis. For the sit-and-reach test, each subject completed 1 practice trial and 2 test trials, and the best score was used. For the 6-minute walking test, 1 test trial was performed. For measurement of walking ability, the neck, elbow, waist, knee, ankle, and sole of the foot of the subjects were marked with colored balls. Then, each subject completed 10 meters of ordinary walking, which was videotaped with a digital video camera recorder (DCR-PC 120, SONY Co., Ltd., Tokyo, Japan) at a sampling rate of 60 Hz. Kinematic analysis of the walking was done using a computerized system. The authors prepared a manual for these measurements beforehand and instructed the examiners (ie, graduate students at Saga University, staff members of the facility, and the fitness instructor) in how to conduct the measurements. The HRQOL measurements were obtained by means of an interview conducted by trained nurses with at least 5 years of experience in a related field.

### Monitoring daily physical activity

A single-axis accelerometer (Kenz Lifecorder EX, Suzuken Co., Ltd., Nagoya, Japan), which measures vertical accelerations at the hip, was used to evaluate daily physical activity. Step outputs from the previous model of the accelerometer have been compared with observed steps in adults; the accuracy of the accelerometer was within 1% to 3%.^22^^,^^23^ The energy expenditure calculated by the accelerometer has also been validated in adults.^24^ The subjects in the intervention group were instructed to wear the accelerometer everyday for 1 year; those in the control group were instructed to wear it every day for the 4 weeks before and after the 12-month intervention period. In the intervention group, one of the authors informed participants of their results bimonthly during their day service visits and encouraged them to walk if the participant had no difficulty doing so.

### Examination of the level of required care

After the 12-month period of intervention, the level of care required for each subject was investigated from the care-level records kept at the 2 facilities. Several subjects were certified to receive Level 2 care (requiring some care in moving, dressing, and daily decision-making, in addition to level 1 care) or level 3 care (requiring full assistance in moving, face washing, dressing, and excretion, in addition to level 2 care), as will be described later. To determine the level of care required, the public long-term care insurance system confirms the computerized assessment by using a standardized interview survey of the physical and mental condition of the insured.^25^ This survey was administered by care managers who were not part of the present study. Each subject in this study was evaluated by the same care manager at baseline and during the intervention period.

### Statistical analysis

The data are expressed as the median (and range, where appropriate). To examine the difference between the 2 groups, we used the Mann-Whitney U test for continuous variables and the chi-square test or Fisher's exact test for categorical variables. The signed rank test was used to compare measurements made before and after the intervention within each group. An odds ratio (plus 95% confidence interval) related to the intervention was estimated in order to examine increases in the required level of long-term care. Statistical significance was defined as *P* < 0.05. The data analysis was completed using the Stat View statistics software package (Version 5.0, SAS Institute Inc., Cary, NC) and SAS for Windows (Version 9.1, SAS Institute Inc.).

## RESULTS

### Attendance, adherence, and pretraining data

Among the 31 subjects in the intervention group and the 34 control subjects who participated in the baseline survey, 25 (81%) intervention subjects and 28 (82%) controls attended the reassessment examination after the 12-month intervention period (Figure 1). Six intervention subjects failed to complete the program because 1 died, 3 were transferred to another facility, and 2 declined. Six control subjects could not participate in the reassessment because 2 were hospitalized, 3 were homebound, and 1 declined (Figure 1). There were no significant differences in baseline data between the individuals who failed to complete the reassessment and those who completed it (data not shown). Furthermore, there were missing data on outcome measures, due to the subjects' poor physical condition, absence from assessment, unwillingness to participate, and measurement error. The respective numbers of intervention group and control group subjects who had measurements before and after the intervention period were 24 and 23 for physical performance, 16 and 17 for walking ability, 21 and 17 for daily physical activity, and 23 and 26 for the HRQOL measurements. The low amount of data on walking ability was mainly due to failures in video recording (eg, out-of-focus recordings) and subject absences.

The attendance rate for the 55 supervised exercise sessions ranged from 54.5% to 100%, with a median of 89.1%. The subjects in the intervention group reported a median of 2.4 home exercise sessions per week. No accidents or medical complications related directly to the exercises were observed.

Table 2 shows the baseline characteristics of the study subjects who attended the baseline survey (including those who could not participate in the reassessment after the 12-month intervention period). The median age was 85 years in the intervention group and 84 years in the control group, and women accounted for 82% of all subjects. No significant differences at baseline were present between the intervention and control groups in anthropometric measurements, level of long-term care required, physical performance, walking ability, daily physical activity, or HRQOL measurements (Table 2).

**Table 2.** Baseline characteristics of study subjects

	Intervention group (*n* = 31)	Control group (*n* = 34)	*P*
Age (years)	85 (74–96)	84 (77–96)	0.45
Male/female (*n*)	6/25	6/28	0.86
Height (cm)	146 (130–172)	146 (134–175)	0.69
Weight (kg)	49.1 (32.8–73.5)	47.5 (35.0–65.5)	0.74
Body mass index (kg/m^2^)	22.2 (16.5–32.7)	22.1 (15.8–29.2)	0.98
Long-term care level (*n*)			
Support required	14	16	0.88
Level 1	17	18	
Physical performance			
Lower-limb strength (kg)	14.5 (5.3–28.0)	13.6 (0–28.0)	0.37
Grip strength (kg)	15.3 (8.0–29.2)	16.4 (8.7–34.8)	0.63
Timed 1-legged standing (sec)	2.0 (0–21.0)	1.5 (0–6.0)	0.43
Sit-and-reach test (cm)	21.0 (3.0–45.0)	25.5 (8.0–50.0)	0.26
6-minute walking distance (m)	240 (41–369)^a^	230 (154–339)	0.51
Walking ability^b^			
Walking speed (m/sec)	0.63 (0.25–1.16)	0.66 (0.29–1.06)	0.38
Stride length (m)	0.35 (0.17–0.52)	0.37 (0.21–0.55)	0.28
Knee joint angle (°)	169 (150–179)	168 (145–176)	0.80
Trunk angle (°)	168 (138–176)	165 (113–176)	0.26
Thigh angle (°)	22.0 (12.9–35.5)	22.9 (13.9–33.8)	0.69
Daily physical activity^c^			
Total steps per day	1068 (233–4691)	914 (134–3218)	0.23
Energy expenditure (kcal/day)	1241 (1005–1690)	1221 (1041–1632)	0.46
Health-related quality of life^d^			
IADL	5.0 (1–7)	4.0 (0–7)	0.18
MMSE	22.0 (14–30)	23.0 (8–28)	0.82
PGC-morale scale	7.0 (2–11)	6.0 (1–11)	0.26
GDS	2.0 (0–4)	3.0 (0–5)	0.24
TMIG-IC	6.5 (0–12)	5.0 (1–12)	0.23
Falls efficacy scale	29.0 (14–37)	28.0 (16–36)	0.50

Values are shown as the median (range) or number. The *P* value was calculated using the Mann-Whitney U test for continuous variables and the χ^2^ test for categorical variables. IADL, Instrumental Activities of Daily Living; MMSE, Mini-Mental State Examination; PGC-morale scale, Philadelphia Geriatric Center Morale Scale; GDS, Geriatric Depression Scale; TMIG-IC, Tokyo Metropolitan Institute of Gerontology Index of Competence.

^a^*n* = 30.

^b^*n* = 25 for the intervention group and 30 for the control group.

^c^*n* = 28 for the intevention group and 31 for the control group.

^d^*n* = 26 for the intevention group and 33 for the control group.

### Change in physical performance, walking ability, daily physical activity, and HRQOL

The baseline measurements of the subjects who had assessments both before and after the intervention period were similar to those shown in Table 2 (data not shown). Table 3 shows the changes in physical performance, walking ability, daily physical activity, and HRQOL in each group after the 12-month intervention period. Regarding physical performance, lower-limb strength and the sit-and-reach test score increased significantly in the intervention group, whereas a significant decrease in grip strength and a marginally significant decrease in 6-minute walking distance (*P* = 0.05) were observed in the control group; each of these changes was significantly different from the corresponding change in the other group. Regarding walking ability, in the control group there were significant decreases in walking speed and stride length, and both these changes significantly differed from the corresponding changes (ie, no significant changes) in the intervention group. Regarding daily physical activity, there were significant decreases in total steps and energy expenditure per day in both the intervention and control groups; however, no significant differences in these decreases were evident between groups. With respect to the HRQOL measurements after the 12-month period, significant changes were observed for the IADL and Falls Efficacy Scale in the intervention group and the MMSE score in the control group. The changes for IADL and MMSE were not significantly different from the corresponding changes in the other group; however, the change in the Falls Efficacy Scale in the intervention group marginally differed (*P* = 0.06) from that in the control group.

**Table 3.** Changes in physical performance, walking ability, daily physical activity, and health-related quality of life in intervention group subjects and control subjects after 12 months of intervention

	Intervention group	Control group	*P*
Physical performance^d^			
Lower-limb strength (kg)	5.0^c^	−1.2	0.004
Grip strength (kg)	−0.2	−2.6^c^	<0.001
Timed 1-legged standing (sec)	0	0.3	0.99
Sit-and-reach test (cm)	8.5^c^	0	<0.001
6-minute walking distance (m)^e^	18.0	−37.0^a^	0.022
Walking ability^f^			
Walking speed (m/sec)	0.15	−0.09^b^	0.005
Stride length (m)	0.06	−0.03^c^	0.002
Knee joint angle (°)	−3.1	−3.6	0.56
Trunk angle (°)	−1.9	−0.8	0.69
Thigh angle (°)	2.7	1.8	0.31
Daily physical activity^g^			
Total steps per day	−269^c^	−205^c^	0.96
Energy expenditure (kcal/day)	−11^b^	−31^b^	0.78
Health-related quality of life^h^			
IADL	1.0^b^	0.5	0.38
MMSE	2.0	1.0^b^	0.75
PGC-morale scale	1.0	0.0	0.44
GDS	0.0	−0.5	0.77
TMIG-IC	0.0	−1.0	0.12
Falls efficacy scale	4.0^c^	−0.5	0.06

Values are shown as the median of individual changes after the intervention period in each group. The *P* value was calculated for the difference between the 2 groups by using the Mann-Whitney U test. IADL, Instrumental Activities of Daily Living; MMSE, Mini-Mental State Examination; PGC-morale scale, Philadelphia Geriatric Center Morale Scale; GDS, Geriatric Depression Scale; TMIG-IC, Tokyo Metropolitan Institute of Gerontology Index of Competence.

^a^*P* = 0.05, ^b^*P* < 0.05, ^c^*P* < 0.01 for the difference between the measurements before and after the intervention period on the signed rank test.

^d^*n* = 24 for the intervention group and 23 for the control group, except for 6-minute walking distance.

^e^*n* = 21 for the intervention group and 21 for the control group.

^f^*n* = 16 for the intervention group and 17 for the control group.

^g^*n* = 21 for the intervention group and 17 for the control group.

^h^*n* = 23 for the intervention group and 26 for the control group.

### Change in level of required long-term care

There was no significant difference between groups in the proportion of subjects who required augmented long-term care during the intervention period (Table 4); the crude odds ratio (plus 95% confidence interval) for an increased need for long-term care associated with the intervention was estimated at 0.56 (0.16–1.89). Although this analysis included the drop-outs who were unavailable for assessment of other outcome measurements, excluding such drop-outs from the analysis did not materially change the results.

**Table 4.** Changes in the level of long-term care required in intervention group subjects and control subjects after 12 months of intervention

Care level	Intervention group (*n* = 30^a^)		Control group (*n* = 34)	
	
	Pre	Post	Pre	Post
Support required	14 (47)	11 (37)	16 (47)	10 (29)
Level 1	16 (53)	18 (60)	18 (53)	20 (59)
Level 2	0	1 (3)	0	3 (9)
Level 3	0	0	0	1 (3)

Change in care level^b^				
Improved	1 (3)		1 (3)	
Unchanged	24 (80)		24 (71)	
Worsened	5 (17)		9 (26)	

Values are shown as the number (percentage).

^a^One of the 31 people in the intervention group died during the intervention period.

^b^The *P* value for the difference in the worsening (as compared to the combined improved and unchanged categories) between the 2 groups was 0.38 on Fisher’s exact test. The crude odds ratio (95% confidence interval) of the worsening associated with the intervention was 0.56 (0.16–1.89).

## DISCUSSION

The main finding of the present study was that, in comparison to the control group, subjects who participated in a 12-month multicomponent exercise program had significant improvements in lower-limb strength and the sit-and-reach test. In addition, grip strength, 6-minute walking distance, walking speed, and stride length significantly decreased in the control group, whereas these measurements remained constant in the intervention group, which suggests that the current exercise program protects against age-related deterioration of these aspects of physical fitness in very elderly people with minor disabilities.

Exercise programs for frail elderly people should be acceptable to and safe for such a population. The average rate of attendance at our supervised exercise program was high (median, 89%), and no injuries were observed in the program. In contrast, a randomized trial of treadmill walking, bicycling, weightlifting, and tai chi training for residents of long-term care facilities had a low adherence rate (mean, 40%) and no treatment effects.^26^ In addition, a high-intensity resistance exercise program led to an increased risk of musculoskeletal injury among frail older people.^27^ Our exercise program did not require any special machines and consisted of multicomponent exercises whose intensity was gradually increased. In addition, all exercises were taught so as to be interactive and enjoyable. These properties may explain the high compliance and safety of this study.

High-intensity strength training has been reported to improve lower-limb strength, even among the old-old.^28^^–^^30^ However, a Japanese study found no significant change in lower-limb strength or flexibility among elderly subjects (median age, 77 years; long-term care Level 1 or 2) after 12 weeks of power rehabilitation using a weight training machine.^31^ It should be noted that few studies have examined the effects of low-intensity, multicomponent, exercise training for very elderly people with physical disabilities or functional limitations.^32^ Worm et al^32^ reported that a 12-week low-intensity, multicomponent, exercise program had a significantly favorable effect on balance, muscle strength, walking function, and self-assessed functional ability in community-dwelling, frail, older people. Similarly, the intervention group in the present study experienced improvements in their lower-limb strength and sit-and-reach test score, even with low-intensity training.

Lower-limb strength and grip strength were inversely related to the risk of death in 75- and 80-year-olds.^33^ In addition, walking speed and stride length were inversely associated with the risk of mortality, dependency, and institutionalization in Chinese elders aged 70 years or older.^34^ A slow walking speed was predictive of the risk of death among 75- and 80-year-olds,^33^ and was associated with the onset of functional dependence in a population of Japanese elders (especially those aged 75 years or older).^35^ We believe that is important to point out that the intervention group in the present study, in comparison to the control group, either improved or maintained their levels of physical performance (ie, lower-limb strength, grip strength, walking speed, and stride length).

One unexpected finding of this study was that daily physical activity significantly decreased in both the intervention and control groups after 12 months. The subjects in this study were very sedentary and seldom went out, as shown by the baseline total steps per day (medians: 1068 and 914 for the intervention and control groups, respectively). Therefore, simply encouraging the intervention group to walk may not have motivated them sufficiently to alter such extremely low activity levels. Moreover, many of the subjects had some form of cognitive impairment, as indicated by the initial MMSE score (medians: 22 and 23 points for the intervention and control groups, respectively),^12^ which may have limited their understanding of the advice regarding walking. Because daily physical activity is known to influence physical fitness and the incidence of certain chronic diseases in older adults,^36^ further studies are required in order to develop intervention methods that are effective in increasing daily physical activity among the old-old.

One of the main goals for an effective program to prevent disability has been improved HRQOL, which appears particularly important for elders receiving nursing care, as they are likely to have lower HRQOL than those not receiving nursing care.^37^^,^^38^ Although several studies have shown beneficial effects of exercise on HRQOL in the very old,^39^^–^^42^ this study failed to demonstrate such an effect for any measure of HRQOL except the Falls Efficacy Scale (*P* for the difference between the two groups = 0.06). A longer follow-up period and a larger sample size may be necessary to examine these effects.

In the revised long-term care insurance system of Japan, preventing a shift in the elderly from minor to moderate or severe disabilities is a major concern. We observed that a multicomponent exercise program may prevent an increase in the need for long-term care in the intervention group subjects (OR = 0.56), although the effect was not statistically significant. This suggests the need for larger studies with longer exercise intervention durations.

There are several limitations in this study. First, the subjects were not randomly allocated to the exercise intervention. This study was conducted under the auspices of the long-term care insurance system, which mandated that the exercise program had to be provided almost equally to all elderly residents of a single facility. This explains why we could not establish a randomized controlled trial design. However, the baseline characteristics of the 2 groups of study subjects were almost identical. Another limitation is that approximately 20% of the subjects dropped out after the baseline survey. Sattin et al^4^ reported that 30% of transitionally frail participants did not complete a 48-week trial, which is a rate similar to that of the present study. Such drop-outs are perhaps inevitable in an intervention trial of the very old. If drop-outs related to the outcome measurements (eg, lower-limb strength) were occurring differentially due to the presence or absence of the exercise intervention, the relevant results may have been biased. However, no material differences in the rate of or reason for drop-outs were observed between the intervention and control groups. Third, in addition to our concerns regarding drop-outs, there were additional missing data on outcome measurements, which could lead to both selection bias and type II errors due to the small sample size. Regarding selection bias, there was no significant difference in the rate of or reason for such missing data between the 2 groups. Due to possible type II errors, however, insignificant results (eg, regarding the Falls efficacy scale) should be accepted with caution. Fourth, the assessors were not blinded to the group assignment. Although we did our best to standardize the testing procedures, as described in the Methods, there could have been some subjective assessments that may have led to measurement bias.

Despite the above limitations, our findings suggest that the present 12-month multicomponent exercise program may improve or maintain physical performance in elderly people with minor disabilities (long-term care Level 1 in the insurance system of Japan). The exercise program required no special training machines, and therefore might be cost-effective for an insurance system whose budget is likely to increase due to the rapidly growing target population.
